# Impact of HIIT Sessions with and without Cognitive Load on Cortical Arousal, Accuracy and Perceived Exertion in Amateur Tennis Players

**DOI:** 10.3390/healthcare10050767

**Published:** 2022-04-21

**Authors:** Vicente Javier Clemente-Suárez, Santos Villafaina, Tomás García-Calvo, Juan Pedro Fuentes-García

**Affiliations:** 1 Faculty of Sports Sciences, Universidad Europea de Madrid, 28670 Madrid, Spain; vctxente@yahoo.es; 2 Grupo de Investigación en Cultura, Educación y Sociedad, Universidad de la Costa, Barranquilla 080002, Colombia; 3 Faculty of Sport Sciences, University of Extremadura, Avenida de la Universidad s/n, 10003 Cáceres, Spain; tgarciac@unex.es (T.G.-C.); jpfuent@unex.es (J.P.F.-G.); 4 Departamento de Desporto e Saúde, Escola de Saúde e Desenvolvimento Humano, Universidade de Évora, 7004-516 Évora, Portugal

**Keywords:** high-intensity interval training, sport, mental load, Stroop

## Abstract

The aim of the present study was to investigate the effects of high-intensity interval training (HIIT) exercises, with and without cognitive load, on the accuracy, critical flicker fusion threshold (CFFT), and rating of perceived exertion (RPE) on recreational tennis players. A total of 32 players of tennis at recreational level (25 men and 7 women) were enrolled in this cross-sectional the study. Participants had to perform, randomly, two HIIT sessions. In one of them, cognitive load was induced by conducting an incongruent Stroop during rests. After training accuracy of tennis serve, CFFT, and RPE were measured. Results showed that accuracy after baseline and HIIT without cognitive load were significantly higher than after HIIT with cognitive load. RPE significantly increased (*p*-value < 0.001) after HIIT sessions in both, with and without cognitive load. However, significant differences were not observed between the two sessions in the RPE (*p*-value = 0.405). Furthermore, differences were not obtained in the CFFT neither within nor between sessions (*p*-value > 0.05). Therefore, HIIT with and without cognitive load increased the RPE in recreational tennis players. Furthermore, HIIT sessions with cognitive load significant altered tennis serve accuracy.

## 1. Introduction

Tennis is an intermittent sport which combines intermittent anaerobic exercise bouts of varying intensities and a multitude of rest periods where players have to perform the technical action with power and accuracy [1]. A previous study indicated that the match result is highly influenced by the “breaks”, or broken services, as well as the percentages of successes to errors, double faults, or errors in returns due to services with good speed and accuracy [2]. Thus, previous studies have analyzed speed [3,4] or accuracy [5] during a tennis serve. Furthermore, participation in recreational tennis may provide benefits in the physical [6,7], psychological, and social spheres [7].

Intermittent exercise has shown to cause a decline in the performance of athletes [8]. The complex phenomenon of fatigue in racquet sports can involve impairments in neural and contractile processes [9]. According to previous studies, this could manifest as mistimed shots, altered on-court movements, and incorrect cognitive choices [10,11,12,13]. In the same line, previous research in the field of tennis showed that fatigue has been shown to reduce the accuracy of returns by 81% [14], groundstrokes by 69% [10], and service by 30% [10]. Therefore, a major goal of tennis training should be to avoid the onset of fatigue during competition and training [1].

The critical flicker fusion threshold (CFFT) has been used to study fatigue and cognitive function in different sport events [15]. This technique is focused on the relationship of arousal level with central nervous system (CNS) [16,17]. When CFFT increases, it suggests an increase in cortical arousal and sensory sensitivity. Nevertheless, when this value decreases, it could mean that the efficiency of the system to process information [18] is reduced and, therefore, could be considered as a symptom of CNS fatigue [19,20].

A previous study [21] investigated the impact of high intensity interval training (HIIT) and intermittent interval training (IIT) on the forehand and backhand shots. Results showed that during the HIIT protocol, the number of errors was significantly higher (a total of 76.20 vs. 51.93 during HIIT and IIT, respectively). Furthermore, tennis performance depends on the interaction between technical, tactical, physiologic, and psychologic skills that often have to be sustained in hostile environmental conditions [9]. Thus, some stressors, such as cognitive load, might be included into training situations in order to simulate real conditions.

However, the impact of conducting HIIT with cognitive load on tennis serve accuracy has not been previously study. This is relevant since, as noted above, tennis players are often under hostile environmental conditions [9], which may cause cognitive load or anxiety. Therefore, the aim of the present study was to investigate the effects of HIIT exercises, with and without cognitive load, on tennis serve accuracy, CFFT, and rating of perceived exertion (RPE) on recreational tennis players. In order to induce cognitive load, an incongruent Stroop test was included during the HIIT rests. This test has been previously used to add cognitive load during physical activities in healthy [22,23] and special populations [24]. The primary hypothesis was that the inclusion of cognitive load in the HIIT session would produce a decrease in cortical arousal and accuracy as well as an increase in the RPE of players. Results and protocols of the present study could be useful for physical trainers and coaches to simulate real conditions (where players have to manage some cognitive stimulus) as well as to design motivating training. Furthermore, results could provide an idea of what happens to serve performance when cognitive load increases.

## 2. Materials and Methods

A total of 32 recreational tennis players (25 men and 7 women) were enrolled in this cross-sectional the study. Participants had a mean age of 21.40 (1.52) and an average experience in tennis practice of 0.84 years (0.80) with 3.26 (0.78) hours of weekly tennis training. The participants weighed an average of 72.18 (11.95) kg, with a mean height of 1.75 (0.8) m and a body mass index mean of 23.48 (2.55). Among the participants, 30 were right-handed and 2 were left-handed. All participants were enrolled in the Faculty of Sport Sciences, Cáceres (Spain).

Procedures were approved by the university ethic committee (approval number: CIPI/18/093), and participants gave written informed consent prior to participation in the study.

### 2.1. Procedures and Materials

**Figure 1:**
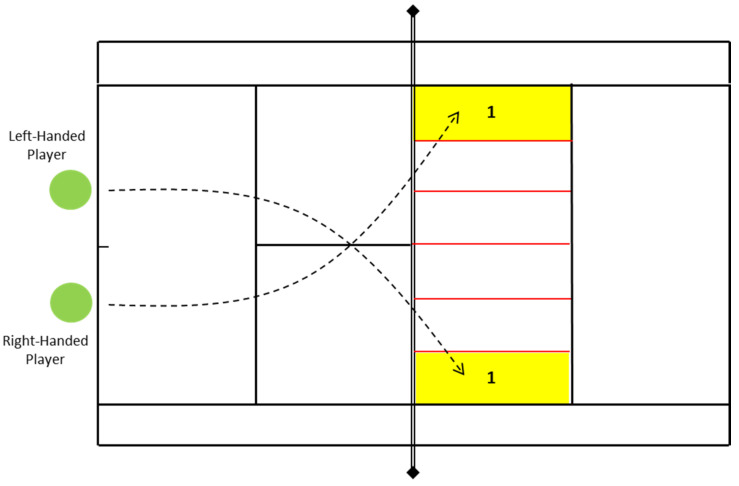
Graphical representation of the area where participants were required to perform the services.

Participants conducted a standardized warm-up composed of 2 min of joint mobility, 5 min of light aerobic running (50−60% of their maximum heart rate calculated with the Tanaka’s Formula “208–0.7 × age” [25] and controlled using the V800, Polar Electro, Kempele, Finland), two series of 20 m of progressive running intensity [26], and five services. Tennis players had to perform seven first services in three different situations: (1) at baseline, (2) after a HIIT training session without cognitive load, and (3) after a HIIT training session with cognitive load. Participants had to serve in the area highlighted in Figure 1 at maximum power. This is an adaptation of a procedure followed by a previous investigation [2].

We assessed if participants were able to serve in this area in either of the two attempts they had. Players were informed about the protocol and service area before starting the procedures.

Two researchers were needed for the data collection, one to provide the ball for the services and another to record if the service impacted in the area. These researchers did not participate in data analyses.

Participants were randomly divided into two groups. Whereas one group started the HIIT session with cognitive load, the other group started the HIIT session without cognitive load. Each of the HIIT sessions (with and without cognitive load) were performed with 48 h of rest between them.

HIIT training sessions consisted of the following:

(1) With cognitive load: Participants had to perform 12 repetitions of 30 s of push-ups, squats, and lateral displacements. After these exercises, participants had to conduct the incongruent Stroop in a validated mobile application (UMH-MEMTRAIN, Elche, Spain) for 30 s. The incongruous condition of the Stroop test consisted of selecting the name of a color, where the color word is printed in an incongruous color ink (i.e., the green word is printed in blue ink). Thus, in this incongruous condition, participants are asked to name the ink color instead of reading the word.
(2) Without cognitive load: 12 repetitions of 30 s of push-ups, squats, and lateral displacements. After these exercises participants rested for 30 s.

### 2.2. Outcomes

The main outcomes of the present study were as follows: (1) The tennis serve accuracy counting with yes/no if participants were able to conduct a tennis serve in the selected area (see Figure 1). (2) The cortical arousal (CFTT) using a Lafayette Instrument Flicker Fusion Control Unit (Model 12021) using the average of 5 incremental test (20 to 100 Hz) as performed in a previous research [27]; and (3) the RPE, on a 6–20 scale [28].

### 2.3. Statistical Analysis

The Statistical Package for the Social Sciences (SPSS) version 25.0 (SPSS Inc., Chicago, IL USA) was used to analyze the data. The Shapiro-Wilk test was conducted to examine the normality of the data. Taking into account the results, non-parametric statistic tests were conducted. The Friedman test was performed to explore the impact of HIIT sessions in the tennis serve accuracy. Moreover, Wilcoxon signed rank tests were performed to explore differences between pre-post assessments in the different sessions as well as to analyze pairwise comparisons in accuracy. In addition, the difference between post and pre values of CFFT and RPE was calculated. This allowed exploration of the impact, through Wilcoxon signed rank tests, of HIIT with cognitive load and HIIT without cognitive load sessions in the CFFT and RPE. The [r] effect size was calculated for Wilcoxon signed rank tests and classified as follows: ≥0.5 is a large effect, 0.5 to 0.1 is a medium effect, and ≤0.1 is considered as a small effect [29,30]. For the Friedman test, the partial η^2^ was calculated and classified as follows: η^2^ = 0.01 indicates a small effect; η^2^ = 0.06 indicates a medium effect; and η^2^ = 0.14 indicates a large effect [31]. The significance level was set at 0.05.

The G*Power software (version 3.1.9.7.) [32] was used to calculate the estimated power. Due to the study design (one group with three measurements), repeated measures ANOVA, within factor statistical test was selected in the G*Power software. This statistical test was chosen because the Friedman test (non-parametric equivalence) was not available in the G*Power software. The means of accuracy values (considered as the main outcome of the study) for baseline, HIIT without cognitive load, and HIIT without cognitive load (see Table 1) and the partial eta squared (effect size) were employed to calculate the statistical power in the G*Power software. Regarding partial eta squared, G*Power automatically transformed it into Cohen’s f effect size. A power equal to 1 (100%) was achieved. The parameters included in the G*Power software were: a total sample size of 32, a Cohen´s f(U) = 2.14 calculated from a 0.814 obtained in the partial eta squared effect size (with effect size specification as in SPSS), an alpha *p*-value = 0.05, one group, three measurements, and a non-sphericity correction of 1.

## 3. Results

Table 1 shows the impact of HIIT training with and without cognitive load in the accuracy of the tennis serve. Friedman Test shows significant differences between the three conditions in the tennis serve accuracy. Pairwise comparisons show that accuracy was significantly higher at baseline than after HIIT with cognitive load (*p*-value = 0.034). Moreover, accuracy after HIIT without cognitive load was significantly higher than after a HIIT with cognitive load (*p*-value = 0.046).

Table 2 shows the impact of HIIT training (with and without cognitive load) on the CFFT and the RPE. Results showed that RPE significantly increased (*p*-value < 0.001) after HIIT sessions for both with and without cognitive load. However, significant differences were not observed between the two sessions in the RPE (*p*-value = 0.405). Furthermore, differences were not obtained in CFFT neither within nor between sessions (*p*-value > 0.05).

## 4. Discussion

This study aimed to investigate the impact of two HIIT sessions, with and without cognitive load, on the tennis serve accuracy, CFFT and RPE on recreational tennis players. Cognitive load was induced by using an incongruent Stroop test during the rests. The primary hypothesis was that the inclusion of cognitive load in the HIIT session would decrease the cortical arousal and the tennis serve accuracy as well as increase the RPE of recreational tennis players. Main findings indicated that accuracy was significantly impacted by HIIT with cognitive load protocol, and that HIIT training (with and without cognitive load) significantly increased CFFT and the RPE.

Results showed that tennis serve accuracy was significantly affected by HIIT sessions, specifically, after a HIIT session with cognitive load. In this regard, it was observed that accuracy decreased from 19.64% in the baseline condition to 17.86% and 11.16% in the HIIT without and with cognitive load, respectively. Furthermore, according to our results, RPE significantly increased after HIIT protocols. Previous studies have reported that fatigue could reduce tennis hitting accuracy of returns by 81% [14], groundstrokes by 69% [10], and service by 30% [10]. Also, in our study it can be observed how accuracy significantly differs between HIIT conditions, showing significantly lower values after HIIT with cognitive load when compared to HIIT without cognitive load. This is consistent with previous studies, which indicated that cognitive load significantly decreased performance [33,34]. This is relevant since tennis performance depends on multiple variable, such as technical, tactical, physiologic, and psychologic skills [9]. In addition, concentrating attention is one of the most important psychological abilities for success in competitive tennis [35]. In relation with that, Pačesová et al. [36] reported that tennis players had better performance on the Stroop test, including in the incongruous condition, where information processing speed, selective attention in the visual system, and inhibitory control are required [37].

Regarding CFFT, results did not show significant changes in cortical arousal after HIIT sessions, with or without cognitive load. Previous studies in military population showed a decrease in cortical arousal [38,39] which has been considered a symptom of fatigue in CNS, reflected by the increase in CFFT values [40]. Furthermore, similar results have been observed after other high intensity activities such as simulated combat or tactical parachute jumps, or even activities with high cognitive requirement [40,41] such as chess [42,43]. A previous investigation hypothesized this could be due to the increase in sympathetic nervous system activation produced in the HIIT that can induce a greater number of cortex efferences to muscles [44].

The RPE showed that both HIIT protocols increased this perception. However, significant changes between the protocols were not observed. The results are similar to those observed in HIIT protocols in military population [38]. Furthermore, previous studies showed that motivation could counteract fatigue-induced performance decrements [45]. However, another study reported that verbal feedback (every 5 s during the 30 s of work) increased intensity, performance, and physical enjoyment during on-court drills [46]. Thus, the inclusion of cognitive elements into HIIT protocols, apart from mimicking real conditions (where players have to manage some cognitive stimulus), can be used as a way to increase motivation during training. In this regard, a previous study in elite youth padel players showed that high levels of motivation could increase players’ mental effort and fatigue during padel training matches [47]. Moreover, HIIT protocols have been used in tennis players in order to improve endurance. Previous research showed that after six weeks of training, the HIIT induced greater improvements in tennis-specific endurance (HIIT 28.9% vs. repeated-sprint ability 14.5%) [48]. In the same line, Kilit and Arslan [49] showed that tennis-specific on-court drills training was more effective in improving agility and technical ability with greater physical enjoyment, whereas HIIT may be more appropriate for speed-based conditioning in younger tennis players. Therefore, cognitive load using incongruous Stroop test could complement on-court training.

This study has some limitations that should be acknowledged. First, the relatively small sample size might cause only the largest differences to reach a level of significance. Second, the sample was comprised of recreational tennis players. This fact means that result extrapolation with elite tennis players or other populations should be made with caution. Third, men and women recreational tennis players were included in the study. However, comparison regarding gender has not been conducted, since only seven females were included. Future studies could explore the differences between genders in the impact of HIIT with cognitive load on accuracy, RPE, and cortisol arousal. Fourth, the accuracy was registered as a binary variable (yes–accurate/no–not accurate). This was selected due to the nature of tennis where if the ball does not land in the area, it is not considered as valid. However, this binary nature does not allow calculation of the variability of the service. Therefore, future studies should explore how cognitive load could influence the variability in precision as previous studies have done with dart-throwing [50]. Despite these limitations, this article has some strengths. For instance, this is the first study exploring the impact of a HIIT session with cognitive load on the accuracy, RPE, and cortical arousal of tennis players. The results will help researchers, coaches, and physical trainers to design sessions that simulate real conditions (in order to improve accuracy in hostile conditions), as well as motivate players. Therefore, future studies and interventions protocols should include activities which combine physical and cognitive activities.

## 5. Conclusions

HIIT with and without cognitive load increased RPE in recreational tennis players. Furthermore, HIIT sessions with cognitive load significantly altered tennis serve accuracy. This is the first study that has examined CFFT after HIIT session with cognitive load in recreational tennis players. The results will help researchers, coaches, and physical trainers to design sessions that simulate real conditions, as well as motivate players. Therefore, future studies and intervention protocols should include activities which combine physical and cognitive activities. Future studies should confirm these results with elite tennis players.

## Figures and Tables

**Table 1:** Effects of HITT sessions, with and without cognitive load, in tennis serve accuracy.

Variable	Baseline	HITT without Cognitive Load Mean (SD)	HITT with Cognitive Load Mean (SD)	Z	*p*-Value	Partial η^2^	Significant Pairwise Comparisons
**Accuracy (%)**	19.64 (16.53)	17.86 (13.58)	11.16 (10.73)	7.828	0.020	0.812	A > C B > C

A: Baseline; B: HITT without cognitive load; C: HITT with cognitive load.

**Table 2:** Impact of HITT sessions, with and without cognitive load, in the CFFT and RPE.

	HITT without Cognitive Load Mean (SD)			HITT with Cognitive Load Mean (SD)			Between Training Comparison		
Variables	Pre	Post	*p*-Value	Pre	Post	*p*-Value	Z	*p*	Effect Size
CFFT (Hz)	34.98 (2.87)	34.26 (3.25)	0.278	33.88 (3.30)	34.15 (3.26)	0.135	−0.701	0.483	0.124
RPE	8.38 (2.22)	14.53 (1.90)	<0.001	9.06 (2.34)	14.44 (2.31)	<0.001	−0.833	0.405	0.147

CFFT: cortical arousal; RPE: Rating of perceived exertion.

## Data Availability

Data are available upon reasonable request to corresponding author.
